# Washed microbiota transplantation improves renal function in patients with renal dysfunction: a retrospective cohort study

**DOI:** 10.1186/s12967-023-04570-0

**Published:** 2023-10-19

**Authors:** Hao-Jie Zhong, Xinqiang Xie, Wen-Jia Chen, Yu-Pei Zhuang, Xuan Hu, Ying-Li Cai, Hong-Lie Zeng, Chuanxing Xiao, Ying Li, Yu Ding, Liang Xue, Moutong Chen, Jumei Zhang, Qingping Wu, Xing-Xiang He

**Affiliations:** 1 Department of Gastroenterology, Research Center for Engineering Techniques of Microbiota-Targeted Therapies of Guangdong Province, The First Affiliated Hospital of Guangdong Pharmaceutical University, Nonglinxia Road 19, Guangzhou, 510000 China; 2 Guangdong Provincial Key Laboratory of Microbial Safety and Health, State Key Laboratory of Applied Microbiology Southern China, Institute of Microbiology, Guangdong Academy of Sciences, Xianliezhong Road 100, Guangzhou, 510000 China; 3 Department of Hepatobiliary and Pancreatic Surgery, First Affiliated Hospital of Shenzhen University, Shenzhen Second People’s Hospital, Shenzhen, China; 4 Department of Oncology, Affiliated Hospital of Nanjing University of Chinese Medicine, Nanjing, China; 5 Guangzhou Treatgut Biotechnology Co., Ltd, Guangzhou, China

**Keywords:** Faecal microbiota transplantation, Chronic kidney disease, Microbiome analysis, Metabolomics analysis, Gut microbiota, Renal insufficiency

## Abstract

**Background:**

Changes in the gut microbiota composition is a hallmark of chronic kidney disease (CKD), and interventions targeting the gut microbiota present a potent approach for CKD treatment. This study aimed to evaluate the efficacy and safety of washed microbiota transplantation (WMT), a modified faecal microbiota transplantation method, on the renal activity of patients with renal dysfunction.

**Methods:**

A comparative analysis of gut microbiota profiles was conducted in patients with renal dysfunction and healthy controls. Furthermore, the efficacy of WMT on renal parameters in patients with renal dysfunction was evaluated, and the changes in gut microbiota and urinary metabolites after WMT treatment were analysed.

**Results:**

Principal coordinate analysis revealed a significant difference in microbial community structure between patients with renal dysfunction and healthy controls (*P* = 0.01). Patients with renal dysfunction who underwent WMT exhibited significant improvement in serum creatinine, estimated glomerular filtration rate, and blood urea nitrogen (all *P* < 0.05) compared with those who did not undergo WMT. The incidence of adverse events associated with WMT treatment was low (2.91%). After WMT, the Shannon index of gut microbiota and the abundance of several probiotic bacteria significantly increased in patients with renal dysfunction, aligning their gut microbiome profiles more closely with those of healthy donors (all *P* < 0.05). Additionally, the urine of patients after WMT demonstrated relatively higher levels of three toxic metabolites, namely hippuric acid, cinnamoylglycine, and indole (all *P* < 0.05).

**Conclusions:**

WMT is a safe and effective method for improving renal function in patients with renal dysfunction by modulating the gut microbiota and promoting toxic metabolite excretion.

**Graphical Abstract:**

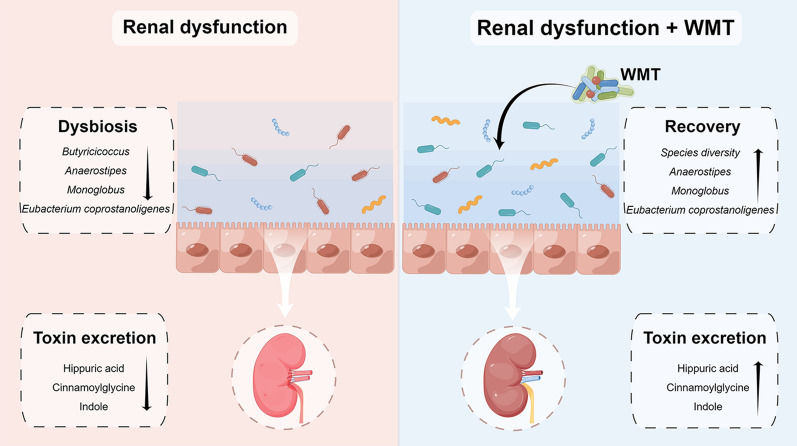

**Supplementary Information:**

The online version contains supplementary material available at 10.1186/s12967-023-04570-0.

## Background

Chronic kidney disease (CKD), affecting approximately 10% of the global population [1, 2], is expected to become the fifth leading cause of death by 2040 [3]. CKD results in a progressive decline in kidney function culminating in end-stage renal disease (ESRD), requiring renal replacement therapy (RRT) for patient survival. The current count of over 2.5 million patients with CKD undergoing RRT is predicted to double, reaching 5.4 million by 2030 [4]. Regrettably, existing therapies offer limited efficacy and only slow disease progression [5]. Consequently, an urgent imperative exists to develop novel approaches that can arrest or reverse the decline in renal function.

Accumulating evidence underscores the involvement of gut microbiota in kidney disease pathophysiology, and a conjectured gut-kidney axis has been proposed [6, 7]. Profound disparities in gut microbiome composition between patients with CKD and healthy controls have been documented [8, 9]. Additionally, animal studies have demonstrated that probiotics, as gut microbiota modulators, can significantly improve renal function in CKD mice [10, 11]. However, in human patients with CKD, probiotics can only delay the decline in renal function rather than effect a cure or reversal [10, 12]. Given the complexity of bacteria-host interactions, a single-species microbiota-targeted intervention might prove insufficient to improve the outcomes of all patients with CKD [13].

Faecal microbiota transplantation (FMT), involving the transfer of multispecies gut microbiota from a healthy donor to a recipient, has proven effective in treating conditions such as *Clostridioides difficile* infection, inflammatory bowel disease, and metabolic disorders [14]. Recent clinical studies have shown FMT’s potential benefits for hypertension, systemic lupus erythematosus, and hyperuricaemia [15–17], which were considered causative factors of CKD. Furthermore, CKD mice treated with healthy-donor gut microbiota exhibited less severe kidney histopathology and lower serum creatinine (SCr) levels compared with those treated with gut microbiota from patients with ESRD [18]. While individual case reports exist [19], no cohort study has addressed whether FMT can improve renal function in patients with renal dysfunction.

Challenges including intricate sample preparation and the high incidence of adverse events (AEs) restrict FMT’s application [20]. Washed microbiota transplantation (WMT), using an automated purification system distinct from traditional FMT, significantly reduces AEs [21]. This study evaluated WMT’s efficacy and safety in improving renal activity among patients with renal dysfunction.

## Methods

### Study design and patients

This retrospective, single-centre, cohort study adhered to the Declaration of Helsinki and obtained approval from the Ethics Committee of the First Affiliated Hospital of Guangdong Pharmaceutical University (approval number: 2021–123). Written informed consent was obtained from all patients, except in cases where a legal representative consented on behalf of those unable to do so.

The study encompassed consecutive adult inpatients (≥ 18 years of age) who underwent WMT and attended at least one follow-up visit at the Department of Gastroenterology, First Affiliated Hospital of Guangdong Pharmaceutical University from 1 January 2017 to 30 June 2021. Additionally, a control group of patients with renal dysfunction, who did not undergo WMT within the same timeframe, was recruited to assess the effect of WMT on renal parameters. The control group was nearly 1:1 matched for sex and age. The exclusion criteria were as follows: (1) acute gastrointestinal infection within 1 month; (2) antibiotic usage within 3 months (except for those who underwent WMT for antibiotic-associated diarrhoea); (3) pregnancy; (4) ongoing RRT (renal transplantation or dialysis) or substantial renal-affecting medication usage (e.g., diuretics or glucocorticoids); and (5) missing medical data. Sample size estimation was performed using online software (Power and Sample Size Calculators; HyLown Consulting LLC, Atlanta, GA, USA).

### Donor selection and WMT procedure

Healthy donors were initially screened using a questionnaire followed by blood and stool tests to rule out communicable diseases, as previously described [15].

A total of 500 mL of 0.9% saline (NaCl) and 100 g of stool sample were homogenised and microfiltered through an automated microbiota purification system (GenFMTer; FMT Medical, Nanjing, China) to prepare the washed microbiota suspension. The faecal microbiota suspension was centrifuged (1100 ×*g* for 3 min at room temperature), and the precipitate was washed with 0.9% NaCl. This process was repeated twice more, each time involving centrifugation and washing. Eventually, 100 mL NaCl was added to resuspend the microbiota precipitate, yielding the final washed microbiota suspension [15].

The WMT procedure involved administering the washed microbiota suspension (120 mL per day for 3 consecutive days) to patients via a transendoscopic enteral tube (for the lower gastrointestinal tract) or a nasojejunal tube (for the upper gastrointestinal tract), according to each patient’s specific conditions and preference. Patients received microbial suspensions from healthy donors, allocated at random.

### Data collection

Electronic medical records provided the following clinical information: demographic details, body mass index, smoking and alcohol habits, history of comorbidities (e.g., hypertension and type 2 diabetes), history of RRT, medication usage, indication for WMT (organic or functional disease), route of WMT delivery (lower or upper gastrointestinal tract), AEs associated with WMT, and laboratory parameters, including SCr, blood urea nitrogen (BUN), serum uric acid (UA), haemoglobin, serum sodium, serum potassium, serum calcium, serum phosphorus, triglycerides, total cholesterol, and low-density lipoprotein cholesterol (LDL-c).

### Definitions

The estimated glomerular filtration rate (eGFR) was calculated as follows: eGFR (mL/min/1.73 m^2^) = 186 × SCr^−1.154^ × age^−0.203^ × (0.742 if female). Normal renal function was defined as eGFR of ≥ 90 mL/min/1.73 m^2^, while renal dysfunction was defined as eGFR of < 90 mL/min/1.73 m^2^ (CKD stages 2–5) [22]. Alcoholism was defined as weekly alcohol consumption of > 210 g for males and > 140 g for females [23]. Organic diseases encompassed conditions resulting in structural changes to the organs or tissues (e.g., inflammatory bowel disease and chronic liver disease), while functional diseases referred to those lacking structural changes (e.g., functional bowel disorders and gut dysbiosis). WMT-related AEs, including abdominal pain, diarrhoea, and fever, were assessed by physicians based on clinical judgment. The effect of WMT on renal parameters was determined as follows: △renal parameter = renal parameter after WMT—renal parameter at baseline.

### Sample collection

Patient stool, urine, and blood samples were collected 2 days before each WMT session (baseline and approximately 1 month, 2 months, and 6 months after the first WMT). Stool samples from healthy donors used for WMT were also collected for sequencing. The stool samples were contained within stool collection tubes with a deoxyribonucleic acid (DNA) stabiliser (Invitek, Germany). All samples were stored at -80℃ until sequencing.

### Microbiome analysis

DNA extraction and sequencing were conducted by Majorbio Bio-Pharm Technology Co. Ltd. (Shanghai, China), as previously described [15]. Briefly, DNA was extracted from each stool sample using the E.Z.N.A.^®^ soil DNA Kit (Omega Bio-Tek, Norcross, GA, USA). DNA concentration was assessed using a NanoDrop 2000 spectrophotometer (Thermo Fisher Scientific, Wilmington, DE, USA). Amplification of bacterial 16S ribosomal ribonucleic acid (rRNA) gene V3–V4 regions was achieved through the 338F and 806R primer sets, and amplicon integrity was verified via agarose gel electrophoresis. Paired-end sequencing was performed using the Illumina MiSeq platform. Raw sequencing reads were deposited in the National Centre for Biotechnology Information Sequence Read Archive under the Accession numbers PRJNA790000.

Paired-end sequences were combined using FLASh (version 1.2.11), and subsequent quality filtering was performed using fastp (version 0.19.6). The remaining sequencing data underwent DADA2-based denoising to generate amplicon sequence variants (ASVs) in QIIME2 (version 2020.2). Taxonomic assignment for the ASVs was performed using QIIME2 and the SILVA 16S rRNA database. Sequencing data analyses were performed using the Majorbio Cloud Platform (www.majorbio.com).

### Metabolomics analysis

For liquid chromatography-mass spectrometry (LC–MS), frozen urine samples were thawed on ice and vortexed. Each urine sample (100 μL) was combined with methanol (300 μL) and 1 μg/mL of L-2-chlorophenyl alanine (Bidepharm, Shanghai, China) as an internal standard for protein precipitation. The mixture was sonicated in an ice-water bath for 10 min, followed by incubation at − 20 ℃ for 1 h and centrifugation at 14,000 ×*g* at 4 ℃ for 15 min. The supernatant (100 μL) was transferred to a glass vial for LC–MS analysis. A quality control sample was prepared by combining 20 μL supernatant from each sample.

LC–MS analysis employed a Q Exactive Plus mass spectrometer (Thermo Fisher Scientific), with all samples analysed in positive and negative ionisation modes. The positive mode mobile phase comprised water with 0.1% formic acid (A) and acetonitrile (B), while the negative mode mobile phase comprised water with 5 mM acetic acid (A) and acetonitrile (B). The column temperature was maintained at 35 ℃, with an injection volume of 3 μL. The gradient elution program was run as follows: 0 min, 1% B; 8 min, 99% B; and 10.1 min, 1% B, at a flow rate of 0.4 mL/min. Electrospray ionisation source parameters included sheath gas flow at 45 L/min, auxiliary gas flow at 15 L/min, sweep gas flow at 0 L/min, spray voltage at 4000 V (for positive mode) or − 3000 V (for negative mode), and capillary temperature at 400 ℃.

Thermo Fisher Scientific Compound Discoverer (version 3.1) facilitated metabolite annotation of LC–MS data, referencing the BioCyc, Human Metabolome, Kyoto Encyclopaedia of Genes and Genomes, MassBank, and National Institute of Standards and Technology databases. Metabolomics analyses and related graphs were generated using MetaboAnalyst 5.0 online tools (www.metaboanalyst.ca). Based on partial least squares discriminant analysis (PLS-DA) results, variable importance in projection (VIP) scores were calculated. Metabolites with VIP scores > 1.0 in the PLS-DA model and *P* < 0.05 in the Wilcoxon rank-sum test were identified as differential metabolites.

### Statistical analysis

Statistical analysis was performed using SPSS software (version 22.0; IBM, Armonk, NY, USA) and Prism (version 8; GraphPad, San Diego, CA, USA). Continuous data are presented as the mean and standard deviation for normally distributed variables and as a median and interquartile range for non-normally distributed variables. Categorical data are presented as frequencies and percentages. Between-group comparisons of continuous variables were performed using the Student’s *t*-test and the Wilcoxon rank-sum test, while categorical variables were analysed using the chi-square test and Fisher’s exact test. For one-sample comparisons (between time points), the one-sample *t*-test or Wilcoxon signed-rank test was used as appropriate. Statistical significance was determined by a two-tailed *P*-value of < 0.05.

## Results

### Demographic characteristics of patients and healthy donors

Initially, 527 patients who underwent WMT were enrolled, and 253 met the final analysis criteria. Of these patients, 86 had renal dysfunction while 168 did not. Among those with renal dysfunction, 76 were in CKD G2, nine in CKD G3, and one in CKD G4. A control group comprising 86 sex- and age-matched patients with renal dysfunction who did not undergo WMT was also included. Additionally, 25 healthy donors passed the donor screening. The demographic and clinical characteristics of patients and healthy donors are summarised in Additional file 3: Table S1.

**Fig. 1:**
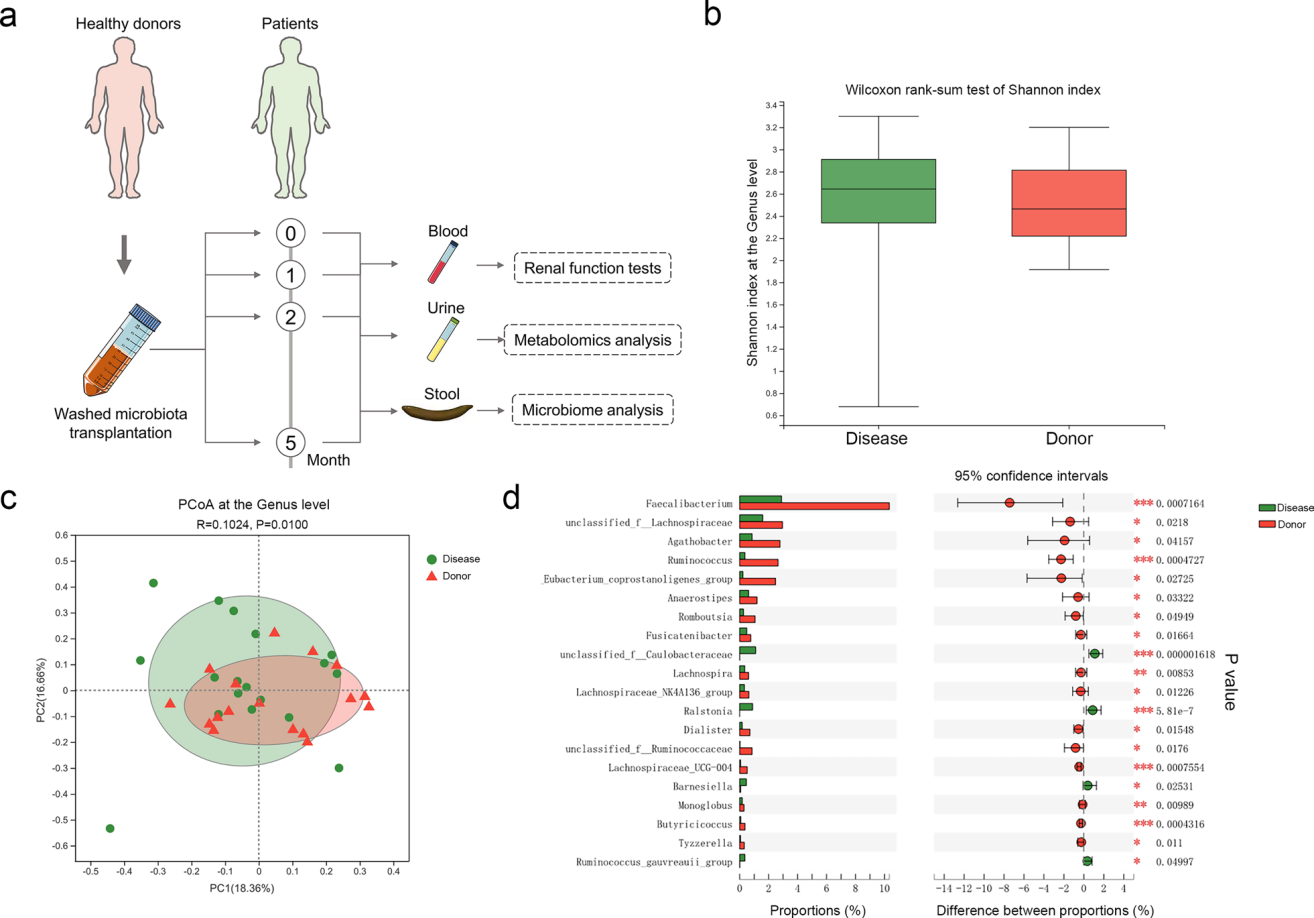
Gut microbiota profiles of patients with renal dysfunction and healthy donors. **a** Study design; **b** Shannon’s diversity index at the genus level; **c** Principal coordinate analysis of microbiota composition at the genus level; **d** Wilcoxon rank-sum test bar plot of relative abundances of the top 20 differential genera. ^*^P < 0.05; ^**^P < 0.01; ^***^P < 0.001

The most prevalent indication for WMT was functional bowel disorders (n = 147), followed by inflammatory bowel disease (n = 32) and chronic liver disease (n = 20; Additional file 4: Table S2). The median intervals between the first and second WMT, second and third WMT, and third and fourth WMT were 36.89 (31.85, 52.00) days, 42.89 (34.03, 64.11), and 97.02 (79.00, 125.03) days, respectively (Fig. 1a).

### Gut microbiota profiles in patients with renal dysfunction and healthy donors

Gut microbiota profiles were compared between patients with renal dysfunction and healthy donors. The phylum-level relative abundances of gut microbes in patients with renal dysfunction and healthy donors are presented in Additional file 1: Fig. S1a. Although no differences were observed in genus-level richness and diversity (Additional file 1: Fig. S1b, Fig. 1b), principal coordinate analysis (PCoA) and nonmetric multidimensional scaling (NMDS) analysis based on β-diversity showed a significantly different microbial community structure between the two groups (Fig. 1c, Additional file 1: Fig. S1c). Compared to healthy donors, patients with renal dysfunction had notable changes in genus-level relative abundances (Fig. 1d, Additional file 1: Fig. S1d). This encompassed reduced relative abundances of *Eubacterium coprostanoligenes*, *Anaerostipes*, *Monoglobus,* and *Butyricicoccus* (all *P* < 0.05).

### Effects of WMT on renal function in patients with or without renal dysfunction

**Fig. 2:**
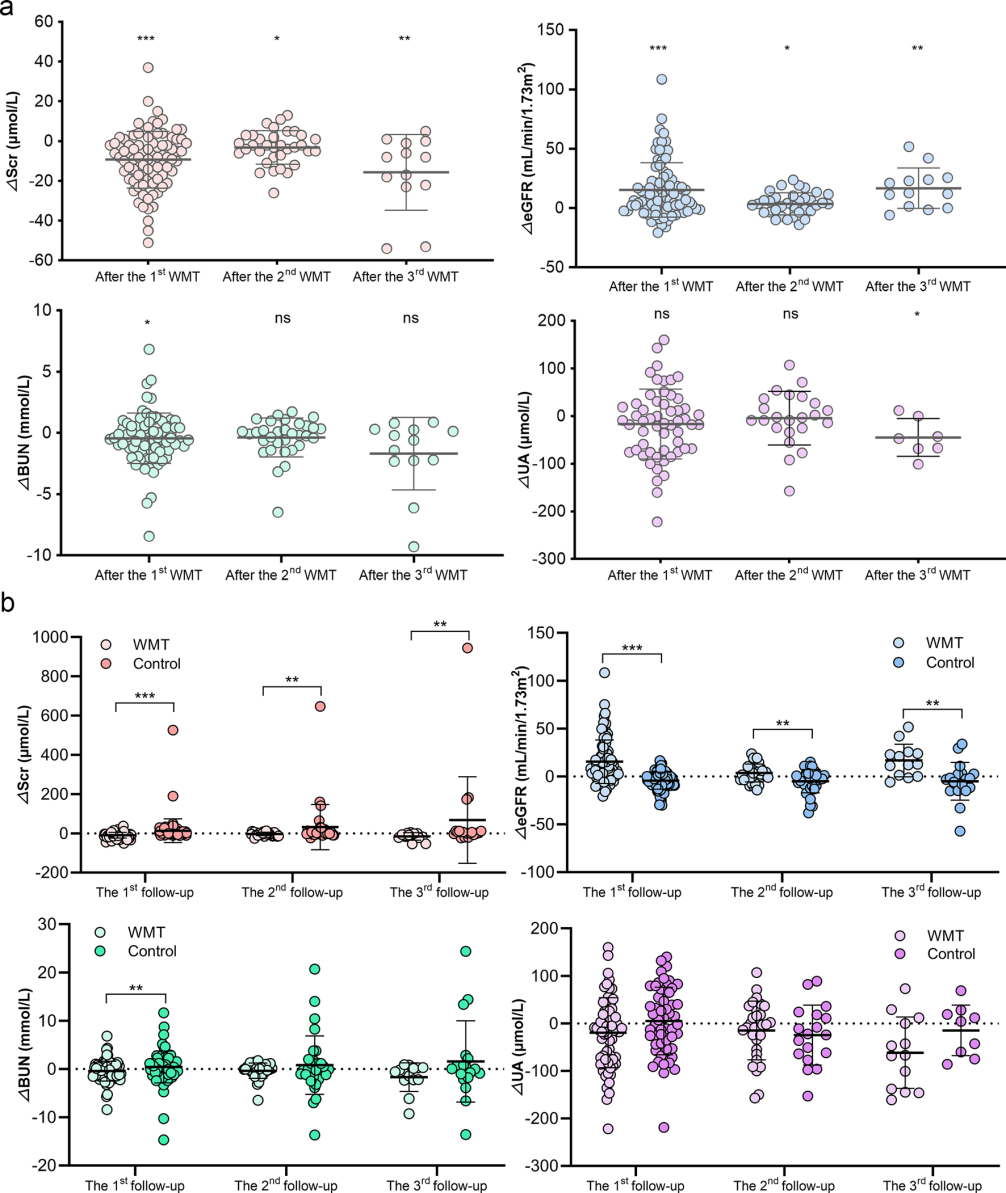
Effects of WMT on renal parameters in patients with renal dysfunction. **a** Changes in the levels of SCr, eGFR, BUN, and UA in patients with renal dysfunction before and after WMT. **b** Comparison of the changes of renal parameters between patients with renal dysfunction who did and did not undergo WMT. △renal parameter = renal parameter after WMT—renal parameter at baseline. BUN, blood urea nitrogen; eGFR, estimated glomerular filtration rate; SCr, serum creatinine; UA, uric acid; WMT, washed microbiota transplantation. ^*^P < 0.05; ^**^P < 0.01; ^***^P < 0.001; ns, no significance

Given the distinctive gut microbiota profiles between patients with renal dysfunction and healthy controls, the study evaluated the influence of gut microbiota remodelling through WMT on renal activity in patients with renal dysfunction. Notably, SCr levels after the first (△SCr: − 9.29 ± 14.31, *P* < 0.01), second (△SCr: − 3.12 ± 8.42, *P* = 0.038), and third (△SCr: − 8.00 [− 22.50, − 0.50], *P* = 0.004) WMT were significantly lower than the levels before WMT, and the eGFR levels after the first (△eGFR: 8.54 [1.02, 23.38], *P* < 0.001), second (△eGFR: 3.58 ± 9.26, *P* = 0.031), and third (△eGFR: 16.72 ± 17.03, *P* = 0.004) WMT were significantly higher than the levels before WMT (Fig. 2a). Additionally, BUN levels after the first WMT (△BUN: − 0.41 [− 1.34, 0.58], *P* = 0.023) and serum UA levels after the third WMT (△UA: − 44.86 ± 39.65, *P* = 0.024) were significantly lower than levels before WMT (Fig. 2a). Furthermore, patients with renal dysfunction who underwent WMT exhibited marked improvements in SCr, eGFR, and BUN compared with those who did not undergo WMT (Fig. 2b).

**Fig. 3:**
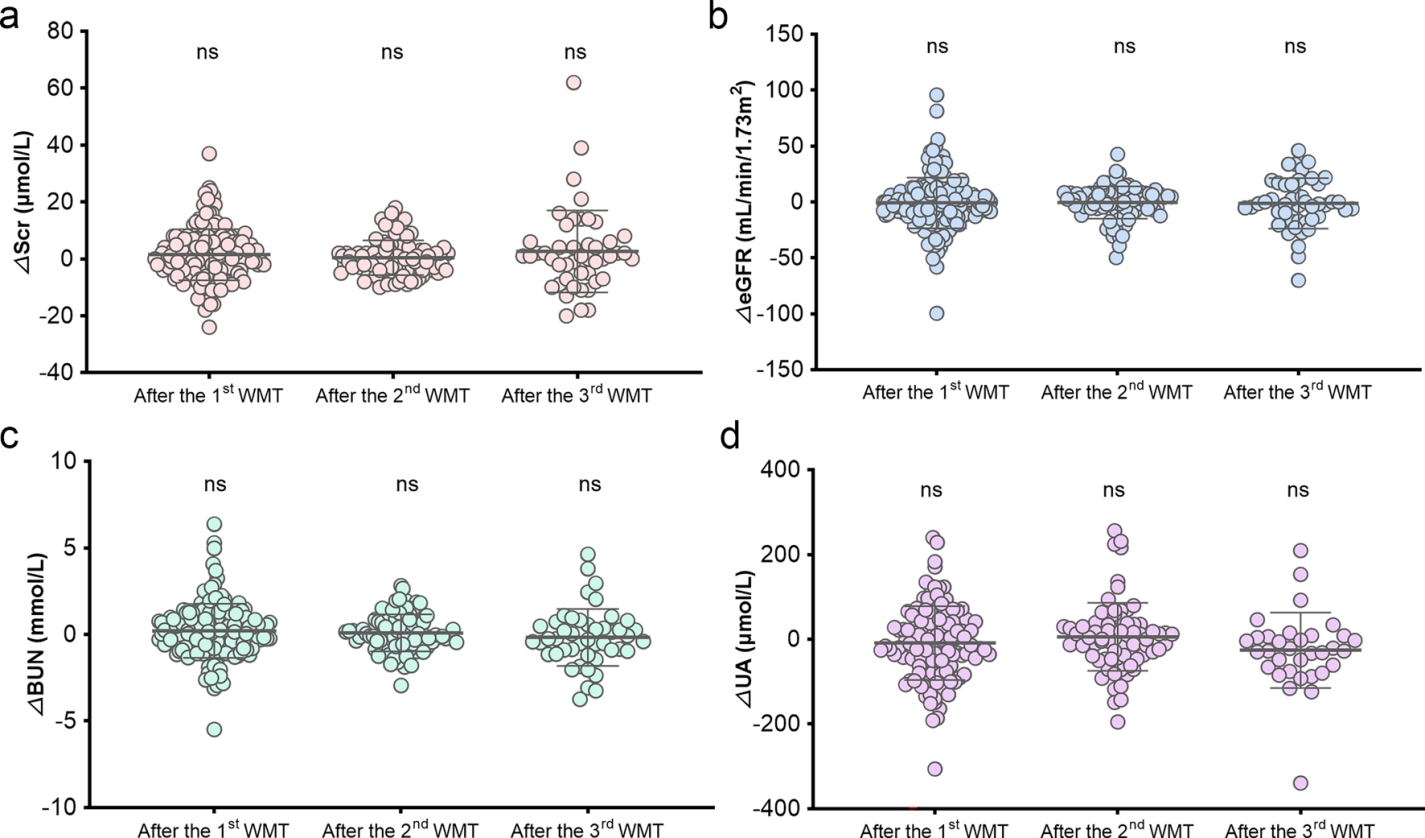
Effects of WMT on renal parameters in patients without renal dysfunction. The effects of WMT on SCr (**a**), eGFR (**b**), BUN (**c**), and UA (**d**) in patients without renal dysfunction. △renal parameter = renal parameter after WMT—renal parameter at baseline. BUN, blood urea nitrogen; eGFR, estimated glomerular filtration rate; SCr, serum creatinine; UA, uric acid; WMT, washed microbiota transplantation. ^*^P < 0.05; ns, no significance

The effects of WMT on renal function in patients without renal dysfunction were also assessed. No significant effects of WMT on renal parameters were observed in these patients, except for a decrease in serum UA after the third WMT (Fig. 3).

### Clinical factors associated with the effects of WMT on renal function

**Fig. 4:**
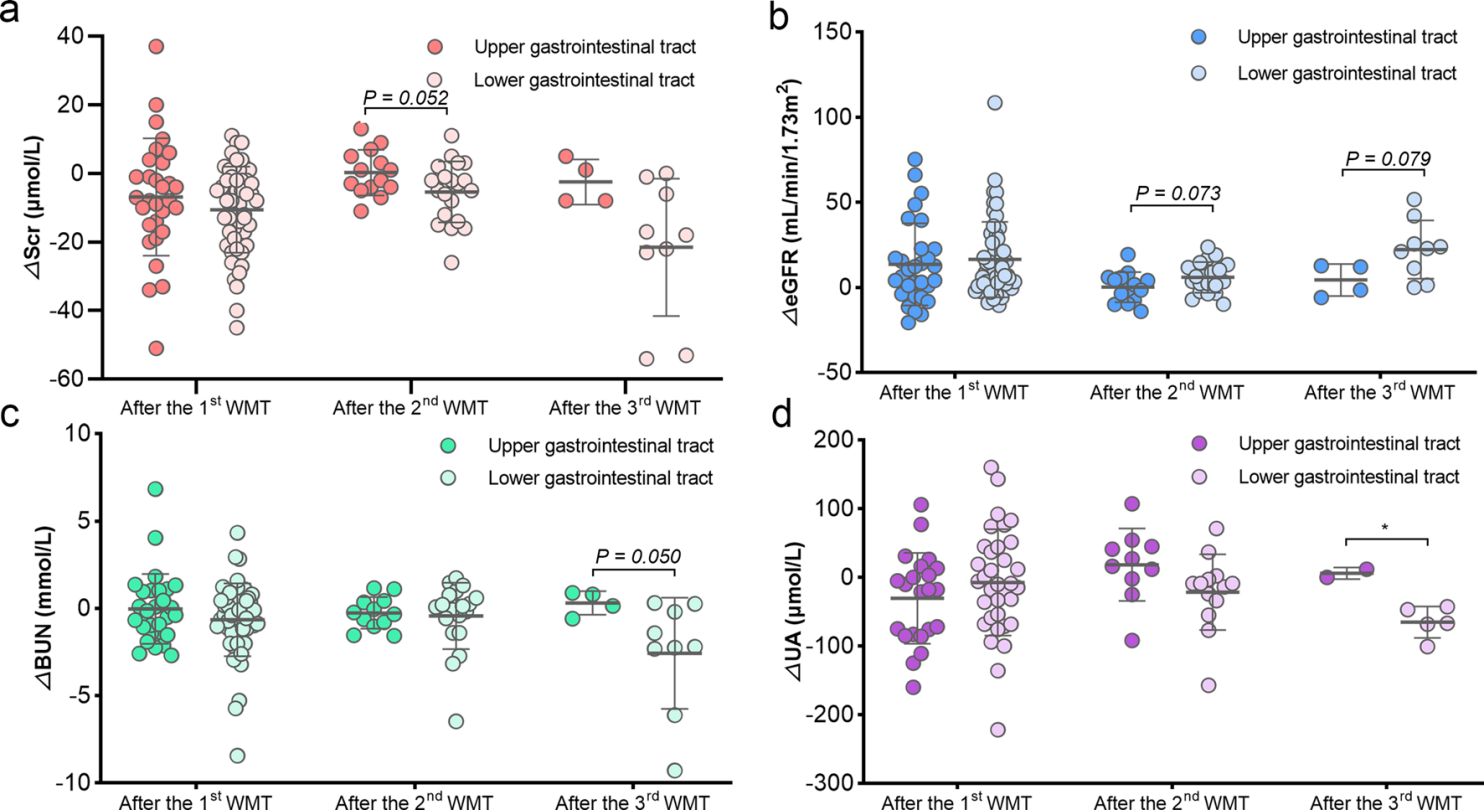
Association between WMT delivery routines and the effects of WMT on renal function. The effects of WMT on SCr (**a**), eGFR (**b**), BUN (**c**), and UA (**d**) in patients with renal dysfunction who underwent WMT through the upper or lower gastrointestinal tract. △renal parameter = renal parameter after WMT—renal parameter at baseline. BUN, blood urea nitrogen; eGFR, estimated glomerular filtration rate; SCr, serum creatinine; UA, uric acid; WMT, washed microbiota transplantation. ^*^P < 0.05

Subsequently, potential factors influencing the effects of WMT on renal function were assessed. Among the patients with renal dysfunction, 56 underwent WMT through the lower gastrointestinal tract, while 30 underwent WMT through the upper gastrointestinal tract. At a significance level of 0.10, the former group displayed greater improvements in SCr after the second WMT (△SCr: − 5.45 ± 8.88 vs. − 0.21 ± 6.65, *P* = 0.052), eGFR after the second (△eGFR: 5.95 ± 8.99 vs. 0.19 ± 8.86, *P* = 0.073) and third (△eGFR: 22.22 ± 17.07 vs. 4.36 ± 9.45, *P* = 0.079) WMT, BUN after the third WMT (△BUN: − 2.20 [− 4.22, 0.03] vs. 0.47 [− 0.41, 0.86], *P* = 0.050), and serum UA after the third WMT (△UA: − 65.20 ± 23.00 vs.6.00 ± 8.49, *P* = 0.010; Fig. 4) compared with the latter group.

**Fig. 5:**
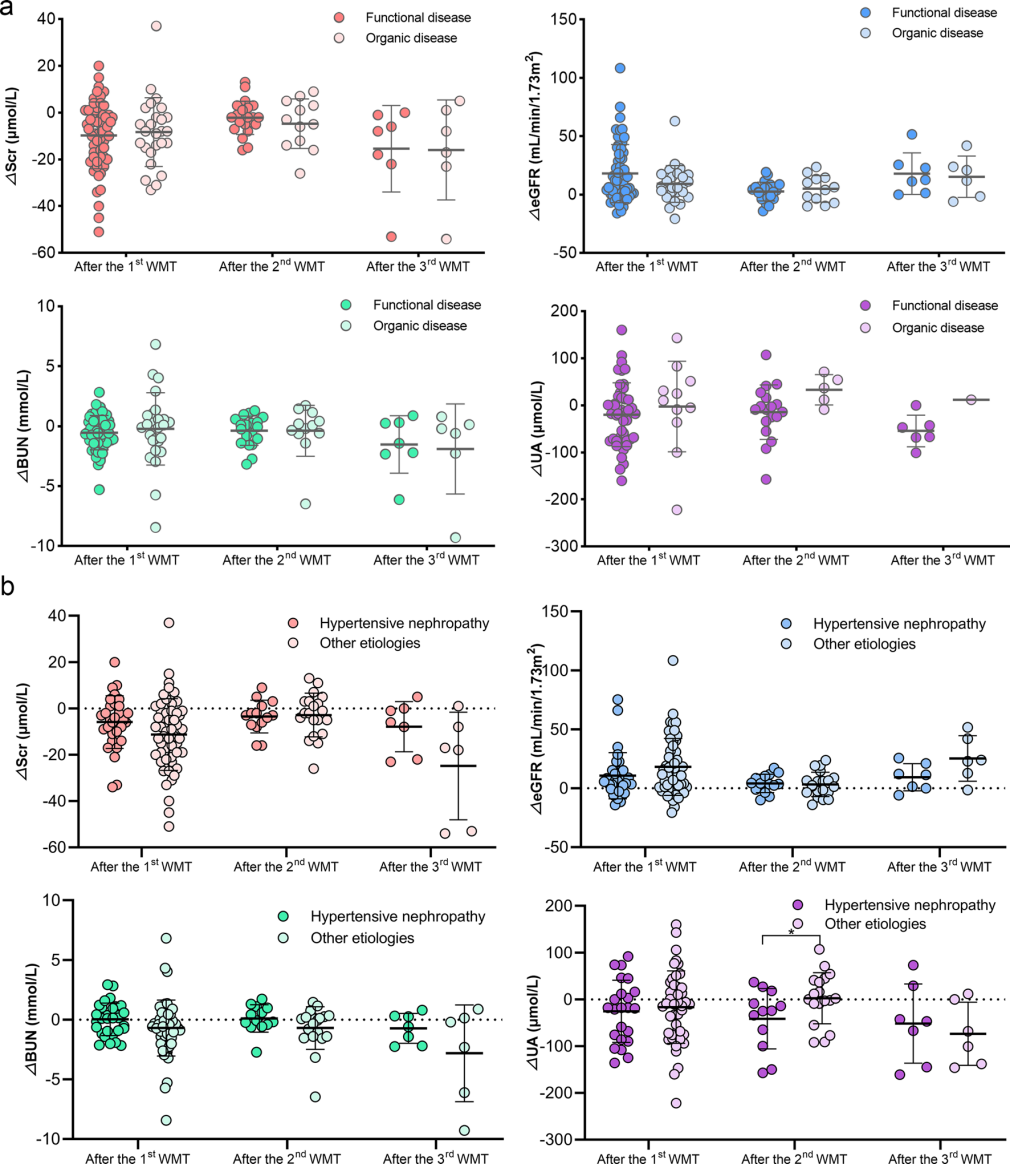
Clinical factors associated with effects of WMT on renal function. **a** Effects of WMT on renal parameters in patients with renal dysfunction who underwent WMT for organic or functional disease; **b** Effects of WMT on renal parameters in patients with renal dysfunction caused by hypertensive nephrology or other aetiologies. △renal parameter = renal parameter after WMT—renal parameter at baseline. BUN, blood urea nitrogen; eGFR, estimated glomerular filtration rate; SCr, serum creatinine; UA, uric acid; WMT, washed microbiota transplantation. ^*^P < 0.05

Among the patients with renal dysfunction, 60 and 26 underwent WMT for functional and organic diseases, respectively. However, no significant differences were observed in the effects of WMT on renal function parameters (SCr, eGFR, BUN, and UA) between these two groups (Fig. 5a). Given hypertensive nephropathy as the primary cause of renal dysfunction, a comparison was drawn between the effects of WMT on renal function parameters in patients with renal dysfunction caused by hypertensive nephrology (n = 31) and those resulting from other aetiologies (n = 55). However, minimal significant differences in most renal function parameters were observed between patients with hypertensive nephropathy and those with other aetiologies (Fig. 5b).

### Effects of WMT on renal disease-related parameters in patients with renal dysfunction

Given that patients with renal dysfunction experience a wide array of complications, including electrolyte disturbances, dyslipidaemia, and anaemia, the impact of WMT on renal disease-related parameters in patients with renal dysfunction was also analysed. The total cholesterol, LDL-c and haemoglobin levels demonstrated signs of improvement after WMT, while other parameters did not exhibit significant changes after treatment (Additional file 5: Table S3).

### AEs of WMT

As safety remains a primary concern in WMT, WMT-related AEs were examined. Among 86 patients with renal dysfunction undergoing 206 WMT procedures, the AE incidence was 2.91%. The most prevalent WMT-related AE was diarrhoea (two WMT procedures, 0.97%), followed by bloating (one WMT procedure, 0.49%), fever (one WMT procedure, 0.49%), vomiting (one WMT procedure, 0.49%), and anal pain (one WMT procedure, 0.49%). Notably, the bloating experienced by one patient resolved spontaneously, while AEs in the remaining five patients improved after symptomatic treatment. No serious AEs were observed.

### Gut microbiota profiles in patients with renal dysfunction before and after WMT

**Fig. 6:**
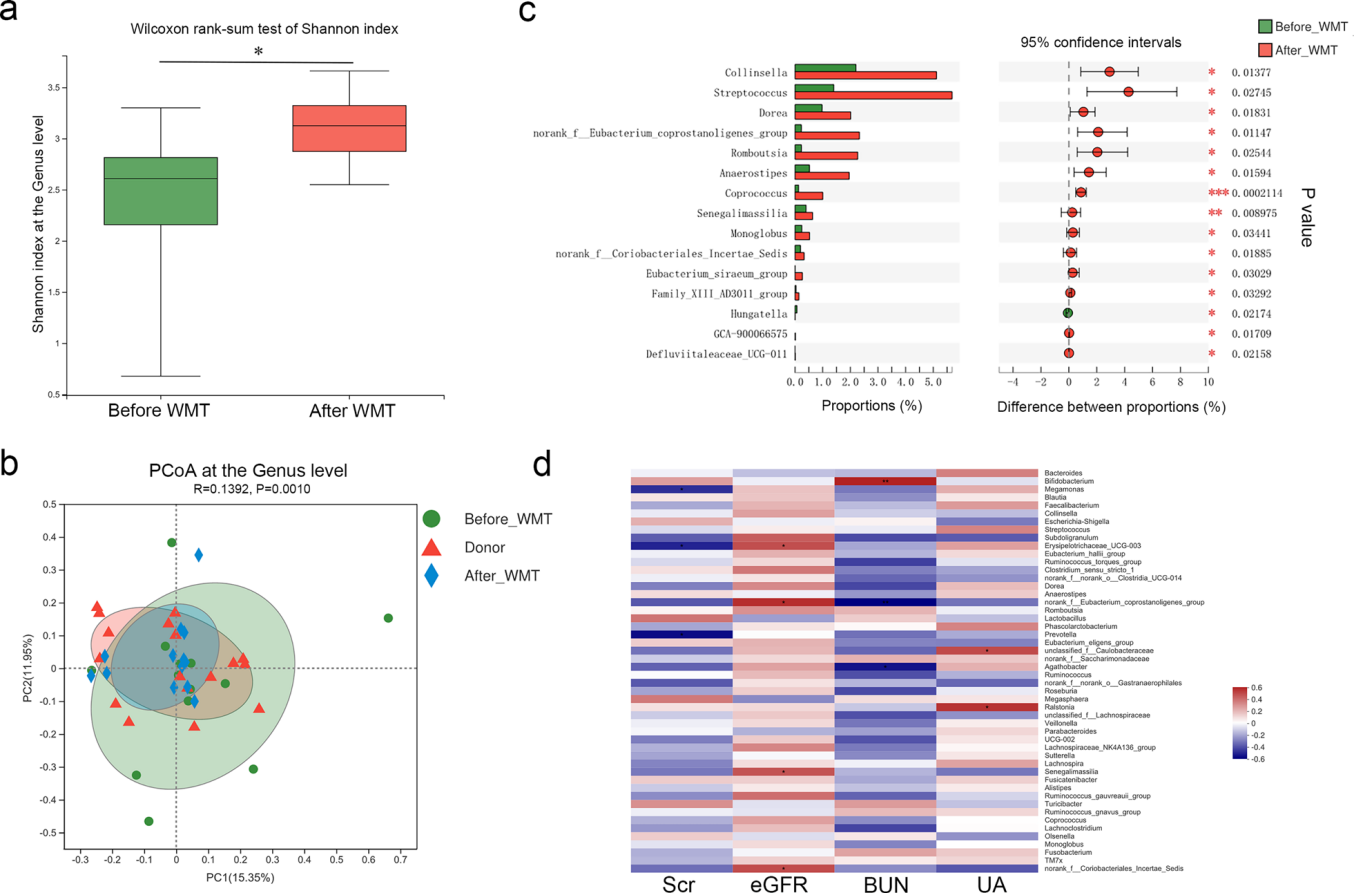
Gut microbiota profiles in patients with renal dysfunction before and after WMT. **a** Shannon’s diversity index at the genus level; **b** Principal coordinate analysis (PCoA) of microbiota composition at the genus level; **c** Wilcoxon rank-sum test bar plot of relative abundances of the top 15 differential genera; **d** Heatmap of the correlations of genus-level abundances and renal parameters. BUN, blood urea nitrogen; eGFR, estimated glomerular filtration rate; SCr, serum creatinine; UA, uric acid; WMT, washed microbiota transplantation. ^*^P < 0.05; ^**^P < 0.01; ^***^P < 0.001

Gut microbiota profiles of patients with renal dysfunction were compared before and after WMT to further investigate the potential mechanism by which WMT improves renal function. A total of 26 stool samples (collected at baseline [n = 13], 1 month [n = 9], 2 months [n = 2], and 6 months [n = 2] after the first WMT) were included for gut microbial analysis. The phylum-level relative abundances of gut microbes in patients with renal dysfunction before and after WMT are presented in Additional file 2: Fig. S2a. The Shannon index (2.32 ± 0.77 vs. 3.09 ± 0.34, *P* = 0.002; Fig. 6a) at the genus level was significantly higher and the Simpson index was significantly lower (0.24 ± 0.22 vs.0.09 ± 0.04, *P* = 0.004; Additional file 2: Fig. S2b) after WMT, while no significant differences were observed in the abundance-based coverage estimator and Chao indices (Additional file 2: Fig. S2b). Genus-level PCoA (R = 0.139, *P* = 0.001; Fig. 6b) and NMDS analysis (stress: 0.264, R = 0.139, *P* = 0.001; Additional file 2: Fig. S2c) demonstrated that the gut microbiota profile of patients with renal dysfunction after WMT tended to resemble that of healthy donors. Notably, several gut genera, including *Eubacterium coprostanoligenes*, *Anaerostipes*, *Monoglobus*, and *Dorea*, exhibited significant enrichment after WMT, having initially been significantly reduced in patients with renal dysfunction. Simultaneously, other genera, including *Hungatella,* were significantly decreased after WMT (Fig. 6c, Additional file 2: Fig. S2d). As presented in Fig. 6d, the relative abundances of several genera correlated with renal parameters in patients with renal dysfunction. For instance, the *Eubacterium coprostanoligenes* group, *Senegalimassilia*, and *Coriobacteriales incertae sedis* abundances were positively correlated with eGFR levels.

### Urine metabolic profiles in patients with renal dysfunction before and after WMT

**Fig. 7:**
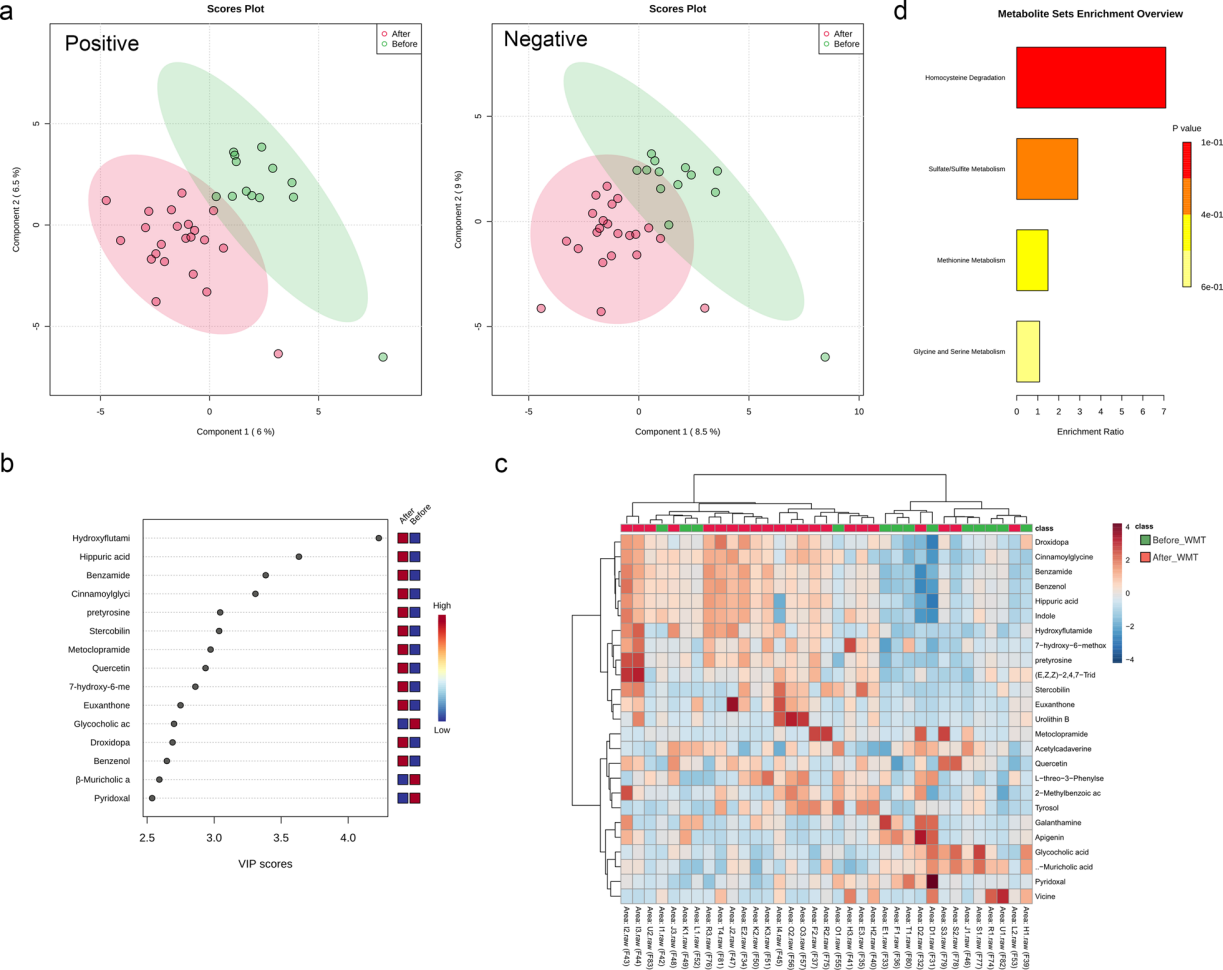
Urine metabolic profiles in patients with renal dysfunction before and after WMT. **a** Partial least squares discriminant analysis (PLS-DA) score plots of the metabolites; **b** Important metabolites identified by PLS-DA based on variable importance in projection (VIP) scores; **c** Heatmap of the abundances of the top 25 metabolites based on the VIP scores; **d** Metabolite set enrichment analysis

Urine metabolomic profiles from 13 patients with renal dysfunction before and after WMT (with available samples at baseline [n = 13], 1 month [n = 12], 2 months [n = 8], and 6 months [n = 2] after the first WMT) were subjected to metabolomics analysis. As demonstrated by the distinct separation in the PLS-DA score plot (Fig. 7a), points representing pre- and post-WMT stages were distinctly separated. VIP scores, derived from PLS-DA outcomes, led to the identification of the top 15 metabolites ranked by VIP scores, as presented in Fig. 7b. Moreover, a heatmap visualised the abundance of the top 25 metabolites based on VIP scores before and after WMT (Fig. 7c). Among these, 16 metabolites with VIP scores > 1.0 and *P* < 0.05 were identified as differential metabolites (Additional file 6: Table S4). More importantly, the relative abundances of three toxic metabolites, namely hippuric acid, cinnamoylglycine, and indole, associated with CKD progression [24–27], were elevated in the urine of patients after WMT (all *P* < 0.05). Using the Small Molecule Pathway Database metabolite set enrichment analysis revealed that pathways such as “homocysteine degradation”, “sulphate/sulphite metabolism”, “methionine metabolism” and “glycine and serine metabolism” experienced notable alterations in patients with renal dysfunction after WMT (Fig. 7d).

## Discussion

**Fig. 8:**
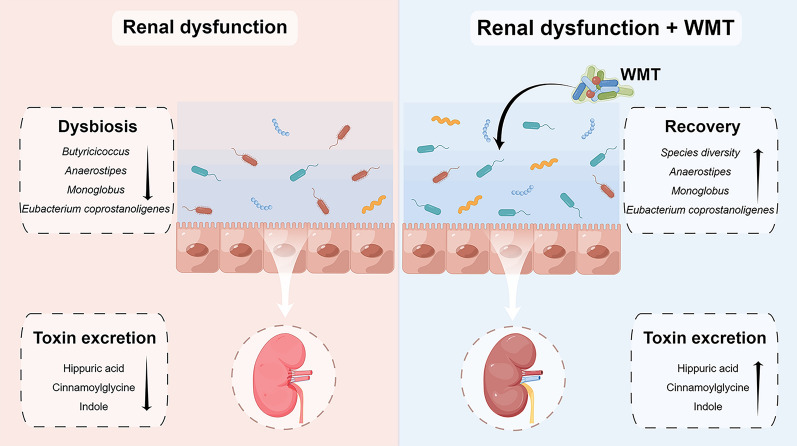
Graphical abstract. WMT, washed microbiota transplantation

This study investigated the efficacy, safety, and underlying mechanism of WMT in enhancing renal activity among patients with renal dysfunction. The findings revealed that WMT resulted in a significant improvement in renal activity for patients with renal dysfunction, while not significantly affecting those without renal dysfunction. In addition, WMT exhibited favourable tolerability and safety, with a low AE incidence (2.91%). After WMT administration, an increase in gut microbiota diversity and the abundance of specific probiotic bacteria were observed in patients with renal dysfunction. Furthermore, their gut microbiota profiles demonstrated a close resemblance to those of healthy donors, and enhanced removal of toxic metabolites through the urine was evident. This suggests that WMT might improve renal function through gut microbiota regulation and improved toxin excretion (Fig. 8). To the best of our knowledge, this is the first clinical study demonstrating the efficacy and safety of WMT in improving renal function in humans.

Current research highlights that patients with CKD have an altered intestinal microbiota [28]. Consistent with previous studies [29, 30], this study reported that the β-diversity of the microbial community was significantly different between patients with renal dysfunction and healthy donors. However, unlike studies finding marked α-diversity variations in mild CKD (CKD stages 1 and 2) compared with patients without CKD, no significant differences in gut microbiota richness or diversity were observed in our study. This aligns with another study suggesting comparable α-diversity in these two patient groups [31]. Furthermore, the study uncovered substantial reductions in the relative abundances of several genera, such as *Eubacterium coprostanoligenes*, *Anaerostipes*, *Monoglobus,* and *Butyricicoccus*, in patients with renal dysfunction compared with healthy donors, which is consistent with previous reports [29, 32–34].

Recent studies have shed new light on the pathogenetic roles of the gut microbiota in kidney diseases, with interventions targeting it (e.g., diet, probiotics, and FMT) holding promise for CKD treatment [13]. Notably, Zhu et al. observed that *Lactobacillus casei* Zhang administration ameliorated gut dysbiosis and slowed disease progression, yet failed to arrest or reverse renal function decline [10]. Likewise, Wang et al*.* demonstrated that healthy donor gut microbiota administration effectively lowered SCr and urea levels, mitigating kidney pathology in CKD mice compared with those receiving microbiota from patients with ESRD, thereby suggesting the potential for FMT to reverse kidney disease progression [18]. In our study, WMT targeted gut microbiota not only arrested but reversed renal function decline among patients with renal dysfunction, suggesting that manipulating gut microbiota might be a novel treatment strategy for CKD.

Several mechanisms could explain these findings. First, patients with CKD exhibit dysbiosis, a change in microbiota composition and structure, with decreased probiotic bacteria and increased pathogenic bacteria [35, 36]. After WMT, the abundances of several probiotic genera, such as *Dorea* and *Anaerostipes*, often reduced in kidney disease [37, 38], increased, while the abundance of potential pathogens such as *Hungatella*, which is significantly increased in patients with CKD [39], markedly decreased in patients with renal dysfunction after receiving WMT. Second, CKD-associated harmful microbiota generates trimethylamine-N-oxide, implicated in uremic toxin accumulation by activating the renin–angiotensin–aldosterone system [40]. Our study evidenced decreased toxic microbiota abundance after WMT, coupled with increased urinary toxin excretion. Therefore, WMT might promote toxin excretion by reducing trimethylamine-N-oxide production, subsequently improving the renin–angiotensin–aldosterone system [19]. Third, uraemia alters the gut biochemical environment, resulting in intestinal mucosal injury (leaky gut), common in CKD. This promotes lipopolysaccharide translocation and serum proinflammatory cytokine production, such as interleukin (IL)-6 and tumour necrosis factor (TNF)-α, exacerbating kidney injury [41, 42]. FMT has been shown to restore intestinal barrier function, lowering serum lipopolysaccharide, IL-6, and TNF-α levels [43], suggesting that WMT may improve renal function by enhancing intestinal barrier integrity and reducing systemic inflammation.

Patients with renal dysfunction who underwent WMT through the lower gastrointestinal tract (with the faecal microbiota suspension reaching the large intestine) experienced more substantial renal function improvement compared with those who received WMT through the upper gastrointestinal tract. Consistent with research on Parkinson’s disease [44] and hypertension [15], colonic FMT demonstrated superiority over nasointestinal FMT. There are two possible explanations for these results. First, location-specific microbes tend to colonise homologous gut regions, suggesting that microbes from the large intestine are more likely to colonise the large intestine than the small intestine [45]. Thus, large-intestine-derived microbes in faecal suspension, when delivered to the large intestine via the lower gastrointestinal tract, might improve microbiota colonisation. Second, patients who received colonic WMT underwent bowel preparation, which potentially facilitated microbiota colonisation, thereby enhancing the therapeutic effect.

Electrolyte abnormalities, dyslipidaemia, and anaemia are common systemic complications of CKD [46]. This study suggested a trend of improvement in blood lipids (total cholesterol and LDL-c) and haemoglobin among patients with renal dysfunction after WMT, indicating the potential of WMT to ameliorate CKD-related metabolic abnormalities and anaemia. Similar observations are seen in clinical studies where FMT increased insulin sensitivity in patients with metabolic syndrome and increased haemoglobin in those with anaemia caused by chronic disease by modulating the intestinal microbiota composition and metabolism [47, 48]. However, whether WMT can improve other CKD-related parameters and complications, such as mineral bone disorder and endocrine dysfunction, remains to be investigated.

This study observed a significant reduction in the abundances of *Eubacterium coprostanoligenes*, *Anaerostipes,* and *Monoglobus* in faecal samples from patients with renal, consistent with findings in patients with immunoglobulin A nephropathy and renal failure [18, 33]. Furthermore, the abundances of *Eubacterium coprostanoligenes*, *Senegalimassilia,* and *Coriobacteriales incertae sedis*, were positively correlated with eGFR levels, indicating their protective role against renal disease progression. Interestingly, WMT led to the abundance of these five genera in patients with renal dysfunction. Further investigation is warranted to assess the therapeutic potential of these genera in CKD management.

Several limitations of our study warrant consideration. First, its retrospective design and small sample size led to a limited number of samples from patients with renal dysfunction. Additionally, the use of DNA stabilising buffer in stool samples posed challenges for metabolomics analysis. Second, several potential confounders, such as protein, water, and salt intake, medication use, and underlying cause of renal dysfunction, which might influence renal disease progression, were incompletely recorded. Third, the relatively short follow-up duration with only 40% and 15% of the patients completing 3 months and 6 months follow-up after WMT, respectively, precludes assessing long-term outcomes of patients with renal dysfunction. Future prospective studies, featuring larger samples and longer follow-up durations are essential to validate these findings.

## Conclusions

In conclusion, WMT proves both safe and effective in improving renal function among patients with renal dysfunction by modulating the gut microbiota and promoting toxic metabolite excretion. These findings suggest that targeting the gut microbiota using WMT offers a promising novel approach for treating CKD.

### Supplementary Information

**Additional file 1: Figure S1.** Gut microbiota profiles of patients with renal dysfunction and healthy donors. **a** Circularised plot of the genus-level abundances in faecal samples; **b** abundance-based coverage estimator (ACE) and Chao and Simpson index at the genus level; **c** nonmetric multidimensional scaling (NMDS) analysis of microbiota composition at the genus level; **d** linear discriminant analysis effect size analysis of the differential genera in stool samples between patients with renal dysfunction and healthy donors.

**Additional file 2: Figure S2.** Gut microbiota profiles of patients with renal dysfunction before and after washed microbiota transplantation (WMT). **a** Bar graph of the genus-level abundances in faecal samples; **b** abundance-based coverage estimator (ACE), Chao and Simpson index at the genus level; **c** nonmetric multidimensional scaling (NMDS) analysis of microbiota composition at the genus level; **d** linear discriminant analysis effect size analysis of the differential genera in stool samples between patients before and after WMT.

**Additional file 3: Table S1.** Demographics and clinical characteristics of the enrolled patients.

**Additional file 4: Table S2.** Reasons for patients undergoing washed microbiota transplantation.

**Additional file 5: Table S3.** Effects of washed microbiota transplantation on renal disease-related parameters in patients with renal dysfunction.

**Additional file 6: Table S4.** Significantly altered metabolites in urine samples from patients before and after washed microbiota transplantation.

**Additional file 7: Table S5.** Untargeted metabolomics data based on the positive mode.

**Additional file 8: Table S6.** Untargeted metabolomics data based on the negative mode.

**Additional file 9: Table S7.** Clinical dataset.

## Data Availability

The data supporting the findings of this study are available at https://dataview.ncbi.nlm.nih.gov/object/PRJNA790000. Untargeted metabolomics data are provided in Additional files 7 and 8.

## Abbreviations

AE: Adverse event
ASV: Amplicon sequence variant
BUN: Blood urea nitrogen
CKD: Chronic kidney disease
DNA: Deoxyribonucleic acid
eGFR: Estimated glomerular filtration rate
ESRD: End-stage renal disease
FMT: Faecal microbiota transplantation
IL: Interleukin
LC–MS: Liquid chromatography-mass spectrometry
LDL-c: Low-density lipoprotein cholesterol
NaCl: Saline
NMDS: Nonmetric multidimensional scaling
PCoA: Principal coordinate analysis
PLS-DA: Partial least squares discriminant analysis
rRNA: Ribosomal ribonucleic acid
RRT: Renal replacement therapy
SCr: Serum creatinine
TNF: Tumour necrosis factor
UA: Uric acid
VIP: Variable importance in projection
WMT: Washed microbiota transplantation

## References

[1] GBD Chronic Kidney Disease Collaboration (2020). Global, regional, and national burden of chronic kidney disease, 1990–2017: a systematic analysis for the Global Burden of Disease Study 2017. Lancet.

[2] Xie Y, Bowe B, Mokdad AH, Xian H, Yan Y, Li T, Maddukuri G, Tsai CY, Floyd T, Al-Aly Z (2018). Analysis of the global burden of disease study highlights the global, regional, and national trends of chronic kidney disease epidemiology from 1990 to 2016. Kidney Int.

[3] Foreman KJ, Marquez N, Dolgert A, Fukutaki K, Fullman N, McGaughey M, Pletcher MA, Smith AE, Tang K, Yuan CW (2018). Forecasting life expectancy, years of life lost, and all-cause and cause-specific mortality for 250 causes of death: reference and alternative scenarios for 2016–40 for 195 countries and territories. Lancet.

[4] Liyanage T, Ninomiya T, Jha V, Neal B, Patrice HM, Okpechi I, Zhao MH, Lv J, Garg AX, Knight J (2015). Worldwide access to treatment for end-stage kidney disease: a systematic review. Lancet.

[5] Ruiz-Ortega M, Rayego-Mateos S, Lamas S, Ortiz A, Rodrigues-Diez RR (2020). Targeting the progression of chronic kidney disease. Nat Rev Nephrol.

[6] Lobel L, Cao YG, Fenn K, Glickman JN, Garrett WS (2020). Diet posttranslationally modifies the mouse gut microbial proteome to modulate renal function. Science.

[7] Zheng DW, Pan P, Chen KW, Fan JX, Li CX, Cheng H, Zhang XZ (2020). An orally delivered microbial cocktail for the removal of nitrogenous metabolic waste in animal models of kidney failure. Nat Biomed Eng.

[8] Wang H, Ainiwaer A, Song Y, Qin L, Peng A, Bao H, Qin H (2023). Perturbed gut microbiome and fecal and serum metabolomes are associated with chronic kidney disease severity. Microbiome.

[9] Hu J, Wei S, Gu Y, Wang Y, Feng Y, Sheng J, Hu L, Gu C, Jiang P, Tian Y (2022). Gut mycobiome in patients with chronic kidney disease was altered and associated with immunological profiles. Front Immunol.

[10] Zhu H, Cao C, Wu Z, Zhang H, Sun Z, Wang M, Xu H, Zhao Z, Wang Y, Pei G (2021). The probiotic *L*. *casei* Zhang slows the progression of acute and chronic kidney disease. Cell Metab..

[11] Kim H, Nam BY, Park J, Song S, Kim WK, Lee K, Nam TW, Park JT, Yoo TH, Kang SW (2022). *Lactobacillus*
*acidophilus* KBL409 reduces kidney fibrosis via immune modulatory effects in mice with chronic kidney disease. Mol Nutr Food Res.

[12] Jia L, Jia Q, Yang J, Jia R, Zhang H (2018). Efficacy of probiotics supplementation on chronic kidney disease: a systematic review and meta-analysis. Kidney Blood Press Res.

[13] Meijers B, Evenepoel P, Anders HJ (2019). Intestinal microbiome and fitness in kidney disease. Nat Rev Nephrol.

[14] Hanssen NMJ, de Vos WM, Nieuwdorp M (2021). Fecal microbiota transplantation in human metabolic diseases: from a murky past to a bright future?. Cell Metab.

[15] Zhong HJ, Zeng HL, Cai YL, Zhuang YP, Liou YL, Wu Q, He XX (2021). Washed microbiota transplantation lowers blood pressure in patients with hypertension. Front Cell Infect Microbiol.

[16] Cai JR, Chen XW, He YJ, Wu B, Zhang M, Wu LH (2022). Washed microbiota transplantation reduces serum uric acid levels in patients with hyperuricaemia. World J Clin Cases.

[17] Huang C, Yi P, Zhu M, Zhou W, Zhang B, Yi X, Long H, Zhang G, Wu H, Tsokos GC (2022). Safety and efficacy of fecal microbiota transplantation for treatment of systemic lupus erythematosus: an EXPLORER trial. J Autoimmun.

[18] Wang X, Yang S, Li S, Zhao L, Hao Y, Qin J, Zhang L, Zhang C, Bian W, Zuo L (2020). Aberrant gut microbiota alters host metabolome and impacts renal failure in humans and rodents. Gut.

[19] Caggiano G, Stasi A, Franzin R, Fiorentino M, Cimmarusti MT, Deleonardis A, Palieri R, Pontrelli P, Gesualdo L (2023). Fecal microbiota transplantation in reducing uremic toxins accumulation in kidney disease: current understanding and future perspectives. Toxins (Basel).

[20] Ding X, Li Q, Li P, Zhang T, Cui B, Ji G, Lu X, Zhang F (2019). Long-term safety and efficacy of fecal microbiota transplant in active ulcerative colitis. Drug Saf.

[21] Zhang T, Lu G, Zhao Z, Liu Y, Shen Q, Li P, Chen Y, Yin H, Wang H, Marcella C (2020). Washed microbiota transplantation vs. manual fecal microbiota transplantation: clinical findings, animal studies and in vitro screening. Protein Cell..

[22] Shlipak MG, Tummalapalli SL, Boulware LE, Grams ME, Ix JH, Jha V, Kengne AP, Madero M, Mihaylova B, Tangri N (2021). The case for early identification and intervention of chronic kidney disease: conclusions from a Kidney Disease: Improving Global Outcomes (KDIGO) Controversies Conference. Kidney Int.

[23] Cai B, Dongiovanni P, Corey KE, Wang X, Shmarakov IO, Zheng Z, Kasikara C, Davra V, Meroni M, Chung RT (2020). Macrophage MerTK promotes liver fibrosis in nonalcoholic steatohepatitis. Cell Metab.

[24] de Boer IH, Gao X, Bebu I, Hoofnagle AN, Lachin JM, Paterson A, Perkins BA, Saenger AK, Steffes MW, Zinman B, Molitch ME (2017). Biomarkers of tubulointerstitial damage and function in type 1 diabetes. BMJ Open Diabetes Res Care.

[25] Gryp T, De Paepe K, Vanholder R, Kerckhof FM, Van Biesen W, Van de Wiele T, Verbeke F, Speeckaert M, Joossens M, Couttenye MM (2020). Gut microbiota generation of protein-bound uremic toxins and related metabolites is not altered at different stages of chronic kidney disease. Kidney Int.

[26] Chen Y, Zelnick LR, Wang K, Hoofnagle AN, Becker JO, Hsu CY, Feldman HI, Mehta RC, Lash JP, Waikar SS (2020). Kidney clearance of secretory solutes is associated with progression of CKD: The CRIC study. J Am Soc Nephrol.

[27] Lim YJ, Sidor NA, Tonial NC, Che A, Urquhart BL (2021). Uremic toxins in the progression of chronic kidney disease and cardiovascular disease: mechanisms and therapeutic targets. Toxins (Basel).

[28] Knauf F, Brewer JR, Flavell RA (2019). Immunity, microbiota and kidney disease. Nat Rev Nephrol.

[29] Ren Z, Fan Y, Li A, Shen Q, Wu J, Ren L, Lu H, Ding S, Ren H, Liu C (2020). Alterations of the human gut microbiome in chronic kidney disease. Adv Sci (Weinh).

[30] Xu KY, Xia GH, Lu JQ, Chen MX, Zhen X, Wang S, You C, Nie J, Zhou HW, Yin J (2017). Impaired renal function and dysbiosis of gut microbiota contribute to increased trimethylamine-N-oxide in chronic kidney disease patients. Sci Rep.

[31] Wu IW, Gao SS, Chou HC, Yang HY, Chang LC, Kuo YL, Dinh MCV, Chung WH, Yang CW, Lai HC (2020). Integrative metagenomic and metabolomic analyses reveal severity-specific signatures of gut microbiota in chronic kidney disease. Theranostics.

[32] Gryp T, Faust K, Van Biesen W, Huys GRB, Verbeke F, Speeckaert M, Raes J, Vaneechoutte M, Joossens M, Glorieux G (2021). Gut microbiome profiling uncovers a lower abundance of Butyricicoccus in advanced stages of chronic kidney disease. J Pers Med.

[33] Chai L, Luo Q, Cai K, Wang K, Xu B (2021). Reduced fecal short-chain fatty acids levels and the relationship with gut microbiota in IgA nephropathy. BMC Nephrol.

[34] Chen TH, Cheng CY, Huang CK, Ho YH, Lin JC (2023). Exploring the relevance between gut microbiota-metabolites profile and chronic kidney disease with distinct pathogenic factor. Microbiol Spectr.

[35] Ondrussek-Sekac M, Navas-Carrillo D, Orenes-Pinero E (2021). Intestinal microbiota alterations in chronic kidney disease and the influence of dietary components. Crit Rev Food Sci Nutr.

[36] Bian J, Liebert A, Bicknell B, Chen XM, Huang C, Pollock CA (2022). Faecal microbiota transplantation and chronic kidney disease. Nutrients.

[37] Yu B, Jin L, Chen Z, Nie W, Chen L, Ma Y, Chen H, Wu Y, Ma Y, Chen J, Han F (2021). The gut microbiome in microscopic polyangiitis with kidney involvement: common and unique alterations, clinical association and values for disease diagnosis and outcome prediction. Ann Transl Med.

[38] Mazidi M, Shekoohi N, Covic A, Mikhailidis DP, Banach M (2020). Adverse impact of Desulfovibrio spp. and beneficial role of Anaerostipes spp. on renal function: insights from a Mendelian randomization analysis. Nutrients..

[39] Hu X, Ouyang S, Xie Y, Gong Z, Du J (2020). Characterizing the gut microbiota in patients with chronic kidney disease. Postgrad Med.

[40] Jiang S, Shui Y, Cui Y, Tang C, Wang X, Qiu X, Hu W, Fei L, Li Y, Zhang S (2021). Gut microbiota dependent trimethylamine N-oxide aggravates angiotensin II-induced hypertension. Redox Biol.

[41] Vaziri ND, Zhao YY, Pahl MV (2016). Altered intestinal microbial flora and impaired epithelial barrier structure and function in CKD: the nature, mechanisms, consequences and potential treatment. Nephrol Dial Transplant.

[42] Yang J, Lim SY, Ko YS, Lee HY, Oh SW, Kim MG, Cho WY, Jo SK (2019). Intestinal barrier disruption and dysregulated mucosal immunity contribute to kidney fibrosis in chronic kidney disease. Nephrol Dial Transplant.

[43] Zhao Z, Ning J, Bao XQ, Shang M, Ma J, Li G, Zhang D (2021). Fecal microbiota transplantation protects rotenone-induced Parkinson's disease mice via suppressing inflammation mediated by the lipopolysaccharide-TLR4 signaling pathway through the microbiota-gut-brain axis. Microbiome.

[44] Xue LJ, Yang XZ, Tong Q, Shen P, Ma SJ, Wu SN, Zheng JL, Wang HG (2020). Fecal microbiota transplantation therapy for Parkinson's disease: a preliminary study. Medicine (Baltimore).

[45] Li N, Zuo B, Huang S, Zeng B, Han D, Li T, Liu T, Wu Z, Wei H, Zhao J, Wang J (2020). Spatial heterogeneity of bacterial colonization across different gut segments following inter-species microbiota transplantation. Microbiome.

[46] Romagnani P, Remuzzi G, Glassock R, Levin A, Jager KJ, Tonelli M, Massy Z, Wanner C, Anders HJ (2017). Chronic kidney disease. Nat Rev Dis Primers.

[47] Kootte RS, Levin E, Salojarvi J, Smits LP, Hartstra AV, Udayappan SD, Hermes G, Bouter KE, Koopen AM, Holst JJ (2017). Improvement of insulin sensitivity after lean donor feces in metabolic syndrome is driven by baseline intestinal microbiota composition. Cell Metab.

[48] Zhong HJ, Chen WR, Lu XJ, Hu DX, Lin DJ, Liu T, Wu L, Wu LH, He XX (2023). Washed microbiota transplantation improves haemoglobin levels in anaemia of chronic disease. Eur J Clin Invest.

