# Supplementary figures and images for: Washed microbiota transplantation improves renal function in patients with renal dysfunction: a retrospective cohort study
Source: J Transl Med. 2023 Oct 19;21:740. doi: 10.1186/s12967-023-04570-0 (PMC10588208; doi:10.1186/s12967-023-04570-0)

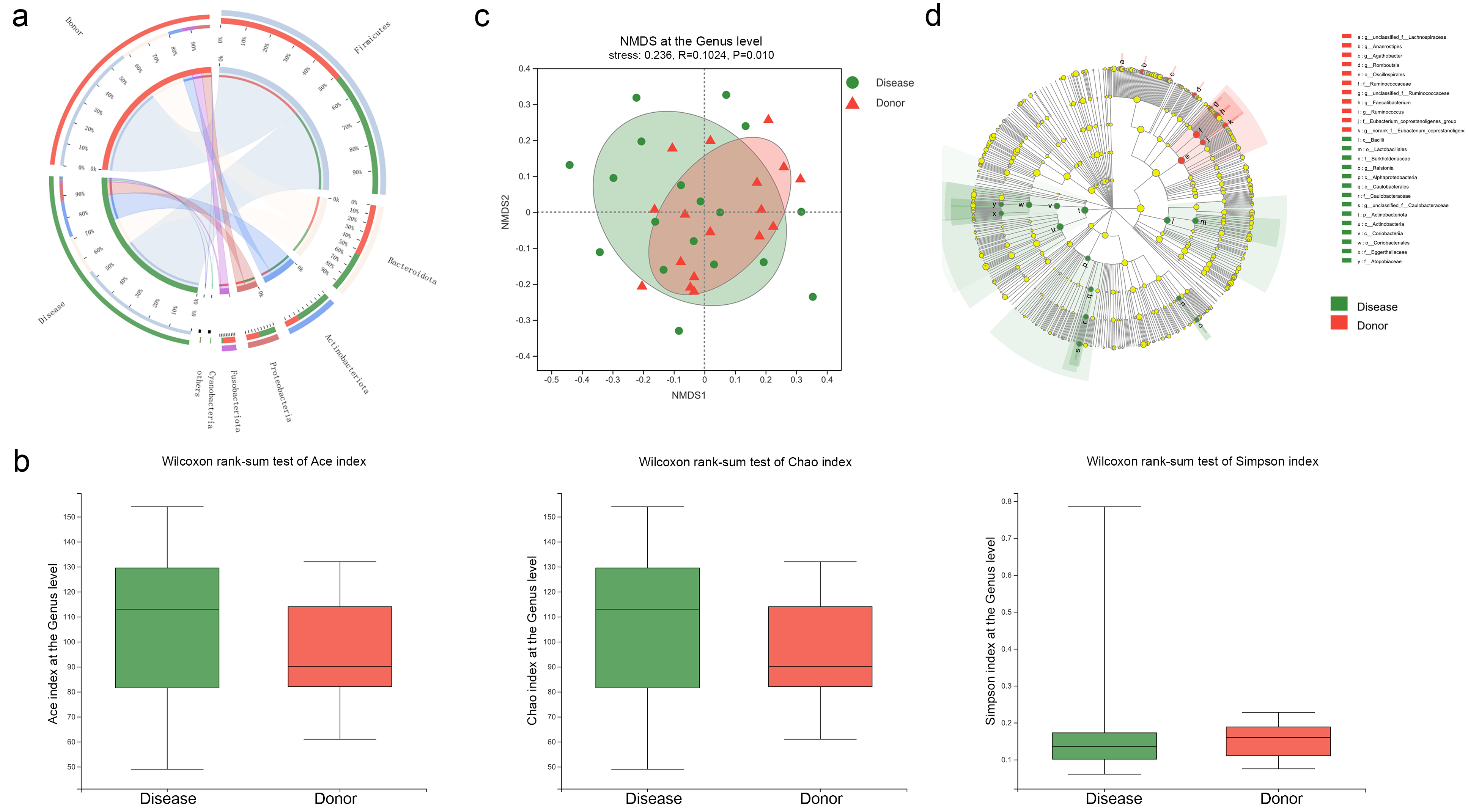

Supplement: Supplementary file 1 — Additional file 1: Figure S1. Gut microbiota profiles of patients with renal dysfunction and healthy donors. a Circularised plot of the genus-level abundances in faecal samples; b abundance-based coverage estimator (ACE) and Chao and Simpson index at the genus level; c nonmetric multidimensional scaling (NMDS) analysis of microbiota composition at the genus level; d linear discriminant analysis effect size analysis of the differential genera in stool samples between patients with renal dysfunction and healthy donors. [file 12967_2023_4570_MOESM1_ESM.tif]

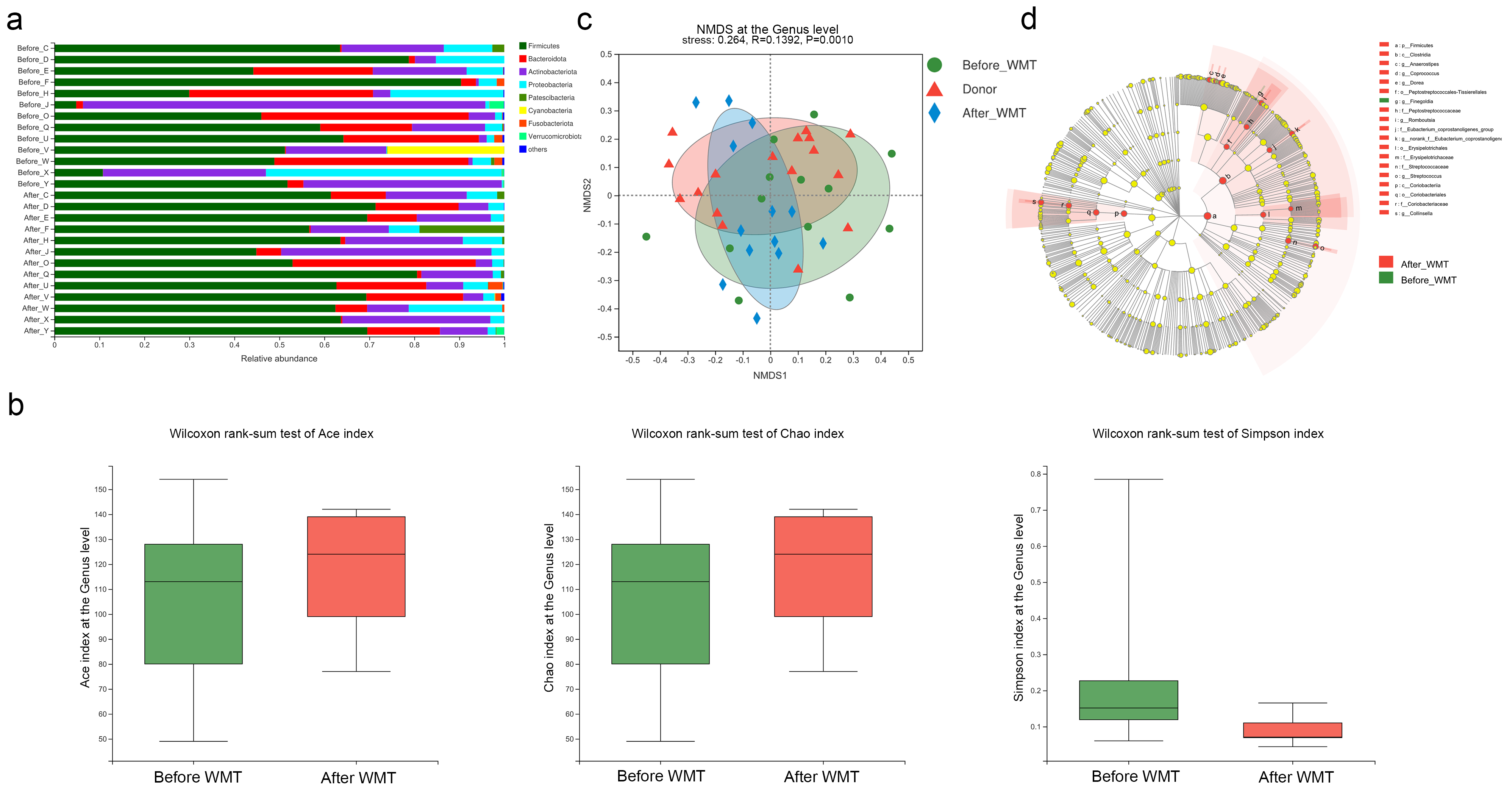

Supplement: Supplementary file 2 — Additional file 2: Figure S2. Gut microbiota profiles of patients with renal dysfunction before and after washed microbiota transplantation (WMT). a Bar graph of the genus-level abundances in faecal samples; b abundance-based coverage estimator (ACE), Chao and Simpson index at the genus level; c nonmetric multidimensional scaling (NMDS) analysis of microbiota composition at the genus level; d linear discriminant analysis effect size analysis of the differential genera in stool samples between patients before and after WMT. [file 12967_2023_4570_MOESM2_ESM.tif]
