# Supplementary material for: Washed microbiota transplantation improves renal function in patients with renal dysfunction: a retrospective cohort study
Source: J Transl Med. 2023 Oct 19;21:740. doi: 10.1186/s12967-023-04570-0 (PMC10588208; doi:10.1186/s12967-023-04570-0)
Supplement: Supplementary file 3 — Additional file 3: Table S1. Demographics and clinical characteristics of the enrolled patients. [file 12967_2023_4570_MOESM3_ESM.docx]

**Table S1. Demographics and clinical characteristics of the enrolled patients and healthy donors.**

|  | Patients with renal dysfunction undergone WMT  n=86 | Patients with renal dysfunction without WMT  n=86 | Patients without renal dysfunction undergone WMT  n=167 | Healthy donors  n=25 |
| --- | --- | --- | --- | --- |
| Male sex | 46 (53.49) | 46 (53.49) | 83 (49.70) | 11(44.00) |
| Age (years) | 64.00 (56.75, 74.00) | 63 (57.00, 74.25) | 49.00 (36.00, 63.00) | 25.00 (23.00, 26.50) |
| Smoking | 14 (16.28) | 15 (17.44) | 28 (16.77) | 0 (0) |
| Alcoholism | 6 (6.98) | 8 (9.30) | 12 (7.19) | 0 (0) |
| Hypertension | 35 (40.70) | 39 (45.35) | 26 (15.57) | 0 (0) |
| Type 2 diabetes | 14 (16.28) | 20 (23.26) | 18 (10.78) | 0 (0) |

WMT, washed microbiota transplantation. Data are presented as n (%) or median (interquartile range).
