# Supplementary material for: Washed microbiota transplantation improves renal function in patients with renal dysfunction: a retrospective cohort study
Source: J Transl Med. 2023 Oct 19;21:740. doi: 10.1186/s12967-023-04570-0 (PMC10588208; doi:10.1186/s12967-023-04570-0)
Supplement: Supplementary file 4 — Additional file 4: Table S2. Reasons for patients undergoing washed microbiota transplantation. [file 12967_2023_4570_MOESM4_ESM.docx]

**Table S2. Reasons for patients undergoing WMT.**

|  | Total  n=253 | Patients with renal dysfunction  n=86 | Patients without renal dysfunction  n=167 |
| --- | --- | --- | --- |
| Functional bowel disorder | 147 | 60 | 87 |
| Inflammatory bowel disease | 32 | 4 | 28 |
| Chronic liver diseases | 20 | 5 | 15 |
| Gastroesophageal disease | 18 | 5 | 13 |
| Carcinoma | 8 | 4 | 4 |
| Gout | 6 | 0 | 6 |
| Radiation enteritis | 5 | 2 | 3 |
| Neuropsychiatric disorder | 4 | 0 | 4 |
| Autoimmune disease | 4 | 0 | 4 |
| Chemotherapy-induced diarrhea | 3 | 3 | 0 |
| Metabolic syndrome | 3 | 2 | 1 |
| Antibiotic-associated diarrhea | 2 | 1 | 1 |
| Intestinal infection | 1 | 0 | 1 |

WMT, washed microbiota transplantation.
