# Supplementary material for: Washed microbiota transplantation improves renal function in patients with renal dysfunction: a retrospective cohort study
Source: J Transl Med. 2023 Oct 19;21:740. doi: 10.1186/s12967-023-04570-0 (PMC10588208; doi:10.1186/s12967-023-04570-0)
Supplement: Supplementary file 5 — Additional file 5: Table S3. Effects of washed microbiota transplantation on renal disease-related parameters in patients with renal dysfunction. [file 12967_2023_4570_MOESM5_ESM.docx]

**Table S3. Effects of WMT on renal disease-related parameters in patients with renal dysfunction.**

|  | WMT | Control | *P* |
| --- | --- | --- | --- |
| △serum sodium |  |  |  |
| The 1^st^ follow-up | 0.00 (-2.00, 1.00)  (n=76) | 0.00 (-1.00, 2.00)  (n=85) | 0.087 |
| The 2^nd^ follow-up | 0.00 (-2.00, 2.25)  (n=30) | -1.00 (-4.00, 2.08)  (n=32) | 0.416 |
| The 3^rd^ follow-up | -0.50 (-2.80, 2.00)  (n=10) | 0.35 (-4.25, 2.23)  (n=18) | 0.953 |
| △serum potassium |  |  |  |
| The 1^st^ follow-up | 0.01 (-0.24, 0.30)  (n=76) | 0.01 (-0.26, 0.20)  (n=85) | 0.759 |
| The 2^nd^ follow-up | 0.02 (-0.28, 0.31)  (n=30) | -0.04 (-0.34, 0.44)  (n=32) | 0.853 |
| The 3^rd^ follow-up | 0.11 (-0.07, 0.25)  (n=10) | -0.11 (-0.44, 0.72)  (n=18) | 0.897 |
| △serum calcium |  |  |  |
| The 1^st^ follow-up | 0.00±0.14  (n=75) | 0.00±0.17  (n=85) | 0.905 |
| The 2^nd^ follow-up | -0.03±0.19  (n=30) | 0.01±0.17  (n=32) | 0.377 |
| The 3^rd^ follow-up | -0.05±0.15  (n=11) | 0.04±0.17  (n=18) | 0.193 |
| △serum phosphorus |  |  |  |
| The 1^st^ follow-up | -0.01±0.15  (n=75) | 0.03±0.18  (n=85) | 0.131 |
| The 2^nd^ follow-up | 0.06 (-0.16, 0.23)  (n=30) | -0.03 (-0.09, 0.15)  (n=32) | 0.597 |
| The 3^rd^ follow-up | -0.01±0.13  (n=11) | -0.01±0.36  (n=18) | 0.972 |
| △total cholesterol |  |  |  |
| The 1^st^ follow-up | -0.15 (-0.54, 0.28)  (n=55) | -0.11 (-0.54, 0.02)  (n=47) | 0.316 |
| The 2^nd^ follow-up | -0.19 (-0.67, -0.02)  (n=25) | -0.05 (-0.67, 0.18)  (n=21) | 0.367 |
| The 3^rd^ follow-up | -0.90±0.69  (n=8) | -0.31±0.70  (n=14) | **0.070** |
| △triglyceride |  |  |  |
| The 1^st^ follow-up | -0.02 (-0.32, 0.14)  (n=55) | 0.08 (-0.36, 0.57)  (n=47) | 0.136 |
| The 2^nd^ follow-up | 0.00 (-0.38, 0.28)  (n=25) | 0.05 (-0.27, 1.16)  (n=21) | 0.252 |
| The 3^rd^ follow-up | 0.10±0.76  (n=8) | -0.04±1.13  (n=14) | 0.773 |
| △low-density lipoprotein-cholesterol |  |  |  |
| The 1^st^ follow-up | -0.11 (-0.46, 0.35)  (n=55) | 0.00 (-0.08, 0.13)  (n=12) | 0.713 |
| The 2^nd^ follow-up | -0.40±0.51  (n=25) | -0.01±0.15  (n=6) | **0.074** |
| The 3^rd^ follow-up | -0.48 (-1.37, -0.28)  (n=8) | 0.18 (0.18, 0.18)  (n=1) | **-** |
| △hemoglobin |  |  |  |
| The 1^st^ follow-up | -1.00 (-5.00, 6.00)  (n=85) | -3.00 (-9.00, 3.50)  (n=86) | **0.060** |
| The 2^nd^ follow-up | -4.00 (-7.50, 6.00)  (n=33) | -6.00 (-11.50, 6.50)  (n=33) | 0.538 |
| The 3^rd^ follow-up | -2.46±8.96  (n=13) | -7.50±20.35  (n=18) | 0.360 |

WMT, washed microbiota transplantation. Data are presented as mean±SD or median (interquartile range).
