# Supplementary material for: Washed microbiota transplantation improves renal function in patients with renal dysfunction: a retrospective cohort study
Source: J Transl Med. 2023 Oct 19;21:740. doi: 10.1186/s12967-023-04570-0 (PMC10588208; doi:10.1186/s12967-023-04570-0)
Supplement: Supplementary file 6 — Additional file 6: Table S4. Significantly altered metabolites in urine samples from patients before and after washed microbiota transplantation. [file 12967_2023_4570_MOESM6_ESM.docx]

**Table S4. Significantly altered metabolites in urine samples from patients before and after WMT.**

|  | VIP | *P* | log2(fold change [after/before WMT]) |
| --- | --- | --- | --- |
| Hydroxyflutamide | 4.2288 | 0.0013426 | 3.516 |
| Hippuric acid | 3.6325 | 0.0033635 | 2.6046 |
| Benzamide | 3.3846 | 0.00075816 | 2.0609 |
| Cinnamoylglycine | 3.3081 | 0.0061195 | 1.8928 |
| Pretyrosine | 3.0448 | 0.00065343 | 1.5933 |
| Quercetin | 2.9357 | 0.011852 | 2.1442 |
| 7-Hydroxy-6-methoxy-2H-chromen-2-one | 2.8596 | 0.0068609 | 1.5106 |
| Droxidopa | 2.6894 | 0.014584 | 1.366 |
| Benzenol | 2.6468 | 0.0013426 | 1.2247 |
| (E,Z,Z)-2,4,7-tridecatrienal | 2.4955 | 0.019679 | 1.8796 |
| Indole | 2.3423 | 0.011852 | 1.0874 |
| Acetylcadaverine | 2.2778 | 0.026202 | -0.88711 |
| 2-Hydroxyhippuric acid | 2.049 | 0.0076791 | -0.14463 |
| Cystathionine | 1.9362 | 0.04472 | -0.70614 |
| Phloretin | 1.9241 | 0.0038051 | -6.9442 |
| Pyrimidine | 1.8205 | 0.041047 | -0.61798 |

VIP, variable importance in projection; WMT, washed microbiota transplantation.
